# Infection fatality rate of COVID-19 inferred from seroprevalence data

**DOI:** 10.2471/BLT.20.265892

**Published:** 2020-10-14

**Authors:** John P A Ioannidis

**Affiliations:** a Meta-Research Innovation Center at Stanford (METRICS), Stanford University, 1265 Welch Road, Stanford, California 94305, United States of America.

## Abstract

**Objective:**

To estimate the infection fatality rate of coronavirus disease 2019 (COVID-19) from seroprevalence data.

**Methods:**

I searched PubMed and preprint servers for COVID-19 seroprevalence studies with a sample size ≥ 500 as of 9 September 2020. I also retrieved additional results of national studies from preliminary press releases and reports. I assessed the studies for design features and seroprevalence estimates. I estimated the infection fatality rate for each study by dividing the cumulative number of COVID-19 deaths by the number of people estimated to be infected in each region. I corrected for the number of immunoglobin (Ig) types tested (IgG, IgM, IgA).

**Findings:**

I included 61 studies (74 estimates) and eight preliminary national estimates. Seroprevalence estimates ranged from 0.02% to 53.40%. Infection fatality rates ranged from 0.00% to 1.63%, corrected values from 0.00% to 1.54%. Across 51 locations, the median COVID-19 infection fatality rate was 0.27% (corrected 0.23%): the rate was 0.09% in locations with COVID-19 population mortality rates less than the global average (< 118 deaths/million), 0.20% in locations with 118–500 COVID-19 deaths/million people and 0.57% in locations with > 500 COVID-19 deaths/million people. In people younger than 70 years, infection fatality rates ranged from 0.00% to 0.31% with crude and corrected medians of 0.05%.

**Conclusion:**

The infection fatality rate of COVID-19 can vary substantially across different locations and this may reflect differences in population age structure and case-mix of infected and deceased patients and other factors. The inferred infection fatality rates tended to be much lower than estimates made earlier in the pandemic.

## Résumé

**Objectif:**

Estimer le ratio de létalité réel de la maladie à coronavirus 2019 (COVID-19) à partir des données de séroprévalence.

**Méthodes:**

J'ai effectué des recherches sur PubMed et sur les serveurs de prépublication afin de trouver des études consacrées à la séroprévalence de la COVID-19, avec des échantillons ≥ 500, au 9 septembre 2020. J'ai également prélevé des résultats supplémentaires dérivés d'études nationales qui figurent dans les versions préliminaires de divers rapports et communiqués de presse. J'ai analysé les études pour y déceler des caractéristiques de conception et des estimations de séroprévalence. Ensuite, j'ai calculé le ratio de létalité réel pour chaque étude en divisant le nombre cumulé de décès dus à la COVID-19 par le nombre d'individus qui auraient été infectés dans chaque région. Enfin, j'ai apporté des corrections en fonction des types d'anticorps testés (immunoglobulines, IgG, IgM, IgA).

**Résultats:**

J'ai pris 61 études en compte (74 estimations) et huit estimations nationales préliminaires. Les estimations en matière de séroprévalence étaient comprises entre 0,02% et 53,40%. Les ratios de létalité réels allaient de 0,00% à 1,63%, les valeurs corrigées de 0,00% à 1,54%. Dans les 51 lieux étudiés, la médiane du ratio de létalité réel pour la COVID-19 s'élevait à 0,27% (0,23% après correction): le ratio était de 0,09% dans les endroits où le taux de mortalité dû à la COVID-19 était inférieur à la moyenne mondiale (< 118 décès/million d'habitants), de 0,20% dans les endroits dénombrant 118–500 décès COVID-19/million d'habitants, et de 0,57% là où la COVID-19 était responsable de > 500 décès/million d'habitants. Chez les personnes de moins de 70 ans, les ratios de létalité réels se situaient entre 0,00% et 0,31% avec des médianes brutes et corrigées de 0,05%.

**Conclusion:**

Le ratio de létalité réel de la COVID-19 peut considérablement varier d'un endroit à l'autre, ce qui pourrait correspondre aux différences de structure de pyramide des âges au sein de la population, au case-mix entre patients infectés et décédés, ainsi qu'à d'autres facteurs. Les ratios de létalité réels que j'ai pu déduire avaient tendance à être nettement inférieurs aux estimations formulées précédemment durant la pandémie.

## Resumen

**Objetivo:**

Estimar la tasa de letalidad por la infección de la enfermedad por coronavirus de 2019 (COVID-19) a partir de los datos de seroprevalencia.

**Métodos:**

Se buscaron los estudios de seroprevalencia de la COVID-19 con un tamaño de muestra mayor o igual a 500 a partir del 9 de septiembre de 2020 en PubMed y en los servidores de preimpresión. Además, se recuperaron los resultados adicionales de los estudios nacionales a partir de los comunicados de prensa y de los informes preliminares. Se evaluaron los estudios para determinar las características de diseño y las estimaciones de seroprevalencia. Para calcular la tasa de letalidad por la infección de cada estudio, se dividió la cantidad acumulada de muertes por la COVID-19 por la cantidad de personas que se estima que están infectadas en cada región. Asimismo, se corrigió la cantidad de tipos de anticuerpos probados (inmunoglobulinas, IgG, IgM, IgA).

**Resultados:**

Se incluyeron 61 estudios (74 estimaciones) y 8 estimaciones nacionales preliminares. Las estimaciones de seroprevalencia oscilaban entre el 0,02 % y el 53,40 %. Las tasas de letalidad por la infección oscilaron entre el 0,00 % y el 1,63 %, los valores corregidos entre el 0,00 % y el 1,54 %. En 51 lugares, la mediana de la tasa de letalidad por la infección de la COVID-19 fue del 0,27 % (corregida en un 0,23 %): la tasa fue del 0,09 % en lugares donde las tasas de letalidad de la población con la COVID-19 eran inferiores al promedio mundial (menos de 118 muertes/millón), del 0,20 % en lugares con 118-500 muertes a causa de la COVID-19/millón de personas y del 0,57 % en lugares con más de 500 muertes a causa de la COVID-19/millón de personas. En personas menores de 70 años, las tasas de letalidad por la infección oscilaron entre el 0,00 % y el 0,31 % con medianas brutas y corregidas del 0,05 %.

**Conclusión:**

La tasa de letalidad por infección de la COVID-19 puede variar de manera sustancial en diferentes lugares y esto puede reflejar diferencias en la estructura de edad de la población y en la variedad de casos de los pacientes infectados y fallecidos, así como en otros factores. Las tasas de letalidad por infección que se calculan tienden a ser mucho más bajas que las estimaciones realizadas a principios de la pandemia.

## ملخص

الغرض تقدير معدل الوفيات الناجمة عن الإصابة بمرض فيروس كورونا 2019 (كوفيد 19) من بيانات الانتشار المصلي.

الطريقة قمت بالبحث في خوادم PubMed وخوادم ما قبل الطباعة عن دراسات الانتشار المصلي لكوفيد 19، بحجم عينة أكبر من أو تساوي 500 بدءاً من 9 سبتمبر/أيلول 2020. كما أنني استرجعت النتائج الإضافية للدراسات الوطنية من البيانات الصحفية والتقارير الأولية. قمت بتقييم دراسات ميزات التصميم وتقديرات الانتشار المصلي. لقد قمت بتقدير معدل الوفيات الناجمة عن الإصابة لكل دراسة عن طريق قسمة العدد الإجمالي للوفيات الناتجة عن جائحة كوفيد 19، على عدد الأشخاص المقدر إصابتهم في كل منطقة. وقمت بتصحيح عدد أنواع الأجسام المضادة التي تم اختبارها (الغلوبين المناعي، IgG ، IgM ، IgA).

النتائج قمت بتضمين 61 دراسة (74 تقديرًا) وثمانية تقديرات وطنية أولية. تراوحت تقديرات الانتشار المصلي من 0.02% إلى 53.40%. تراوحت معدلات وفيات العدوى من 0.00% إلى 1.63%، وتم تصحيح القيم من 0.00% إلى 1.54%. عبر 51 موقعًا، كان متوسط ​​معدل وفيات عدوى كوفيد 19 هو 0.27% (تصحيح بنسبة 0.23%): كان المعدل 0.09% في المواقع التي تقل فيها معدلات وفيات السكان المصابين بكوفيد 19 عن المتوسط ​​العالمي (أكثر من 118 حالة وفاة/مليون نسمة)، و0.20% في المواقع التي يوجد بها من 118 إلى 500 حالة وفاة/مليون نسمة مصابين بكوفيد 19، و0.57% في مواقع بها أكثر من 500 حالة وفاة/مليون نسمة بسبب كوفيد 19. في الأشخاص الذين تقل أعمارهم عن 70 عامًا، تراوحت معدلات وفيات الإصابة بالعدوى من 0.00% إلى 0.31%  بمتوسطات مبدئية ومصححة قدرها 0.05%.

الاستنتاج يمكن أن يختلف معدل وفيات الإصابة بفيروس كوفيد 19 بشكل كبير عبر المواقع المختلفة، وقد يعكس هذا الاختلافات في التركيب العمري للسكان، ومزيج الحالات من المرضى المصابين والمتوفين، وعوامل أخرى. تميل معدلات الوفيات المستدل عنها من العدوى إلى أن تكون أقل بكثير من التقديرات التي تم إجراؤها في وقت سابق في الجائحة.

## 摘要

**目的:**

根据血清阳性率数据估计 2019 年冠状病毒病（新型冠状病毒肺炎）的感染死亡率。

**方法:**

在 PubMed 和预印本服务器上查找截至 2020 年 9 月 9 日新型冠状病毒肺炎相关的血清阳性率研究，样本量为 500 个。另外根据初步新闻稿和报告检索了其他全国性研究结果。并评估了与设计特征和血清阳性率估计值相关的研究。通过将新型冠状病毒肺炎累计死亡人数除以每个地区估计感染人数，估算出了每项研究的感染死亡率。然后校正了测试的抗体类型（免疫球蛋白、免疫球蛋白 G、免疫球蛋白 M、免疫球蛋白 A）的数量。

**结果:**

我汇总了 61 项研究（74 个估计值）和 8 个全国性初步估计值。血清阳性率估计值介于 0.02％ 至 53.40％ 之间。感染死亡率介于 0.00％ 至 1.63％ 之间，校正值则介于 0.00％ 至 1.54％ 之间。在 51 个地区中，新型冠状病毒肺炎感染死亡率的中位数为 0.27％（校正值为 0.23％）：在新型冠状病毒肺炎导致的人口死亡率低于全球平均水平（每一百万人口中死亡病例小于 118 例）的地区中，该比率为 0.09％；在每一百万人口中新型冠状病毒肺炎死亡病例介于 118–500 例之间的地区，该比率为 0.20％；而在每一百万人口中新型冠状病毒肺炎死亡病例大于 500 例的地区，该比率则为 0.57％。70 岁以下人群的感染死亡率介于 0.00％ 至 0.31％ 之间，经粗略校正后该比率的中位数为 0.05％。

**结论:**

不同地区的新型冠状病毒肺炎感染死亡率可能存在很大的差异，据此可反映出在人口年龄结构、感染和死亡病例组合以及其他因素方面存在差异。推断的感染死亡率往往比全球性流行病爆发初期的估计值要低得多。

## Резюме

**Цель:**

Оценить показатель летальности при инфицировании коронавирусным заболеванием 2019 г. (COVID-19) на основании данных о серораспространенности.

**Методы:**

Автор провел поиск на серверах PubMed и серверах предварительной публикации на предмет исследований серораспространенности COVID-19 с размером выборки ≥500 по состоянию на 9 сентября 2020 года. Были также получены дополнительные результаты национальных исследований из предварительных пресс-релизов и отчетов. Автор оценил исследования по элементам дизайна и оценкам серораспространенности. Автор оценил показатель летальности при инфицировании для каждого исследования, разделив общее количество смертей от COVID-19 на количество людей, предположительно инфицированных в каждом регионе. При этом была сделана поправка на количество протестированных типов антител (иммуноглобины, IgG, IgM, IgA).

**Результаты:**

В работу вошло 61 исследование (74 прогноза) и восемь предварительных национальных прогнозов. Прогнозы серораспространенности варьировались в диапазоне от 0,02 до 53,40%. Показатели летальности при инфицировании варьировались в диапазоне от 0,00 до 1,63%, скорректированные значения — от 0,00 до 1,54%. В 51 регионе средний показатель летальности при инфицировании COVID-19 составил 0,27% (скорректированный показатель 0,23%): этот показатель составил 0,09% в регионах с уровнем летальности населения от COVID-19 ниже, чем в среднем по миру (<118 смертей на миллион), 0,20% в регионах, в которых зарегистрировано 118–500 случаев смерти от COVID-19 на миллион человек, и 0,57% в регионах, где зарегистрировано более 500 случаев смерти от COVID-19 на миллион человек. У людей младше 70 лет показатель летальности при инфицировании колебался в пределах от 0,00 до 0,31% с приблизительными и скорректированными медианными значениями 0,05%.

**Вывод:**

Показатель летальности при инфицировании COVID-19 может существенно различаться в разных регионах, и это может отражать различия в возрастной структуре населения, структуре случаев инфицирования и смерти пациентов, а также в других факторах. Предполагаемые показатели летальности при инфицировании, как правило, были намного ниже, чем прогнозы, сделанные ранее во время пандемии.

## Introduction

The infection fatality rate, the probability of dying for a person who is infected, is one of the most important features of the coronavirus disease 2019 (COVID-19) pandemic. The expected total mortality burden of COVID-19 is directly related to the infection fatality rate. Moreover, justification for various non-pharmacological public health interventions depends on the infection fatality rate. Some stringent interventions that potentially also result in more noticeable collateral harms^1^ may be considered appropriate, if the infection fatality rate is high. Conversely, the same measures may fall short of acceptable risk–benefit thresholds, if the infection fatality rate is low.

Early data from China suggested a 3.4% case fatality rate^2^ and that asymptomatic infections were uncommon,^3^ thus the case fatality rate and infection fatality rate would be about the same. Mathematical models have suggested that 40–81% of the world population could be infected,^4^^,^^5^ and have lowered the infection fatality rate to 1.0% or 0.9%.^5^^,^^6^ Since March 2020, many studies have estimated the spread of the virus causing COVID-19 – severe acute respiratory syndrome coronavirus 2 (SARS-CoV-2) – in various locations by evaluating seroprevalence. I used the prevalence data from these studies to infer estimates of the COVID-19 infection fatality rate.

## Methods

### Seroprevalence studies

The input data for calculations of infection fatality rate were studies on the seroprevalence of COVID-19 done in the general population, or in samples that might approximately represent the general population (e.g. with proper reweighting), that had been published in peer-reviewed journals or as preprints (irrespective of language) as of 9 September 2020. I considered only studies with at least 500 assessed samples because smaller data sets would result in large uncertainty for any calculations based on these data. I included studies that made seroprevalence assessments at different time intervals if at least one time interval assessment had a sample size of at least 500 participants. If there were different eligible time intervals, I selected the one with the highest seroprevalence, since seroprevalence may decrease over time as antibody titres decrease. I excluded studies with data collected for more than a month that could not be broken into at least one eligible time interval less than one month duration because it would not be possible to estimate a point seroprevalence reliably. Studies were eligible regardless of the exact age range of participants included, but I excluded studies with only children.

I also examined results from national studies from preliminary press releases and reports whenever a country had no other data presented in published papers or preprints. This inclusion allowed these countries to be represented, but information was less complete than information in published papers or preprints and thus requires caution.

I included studies on blood donors, although they may underestimate seroprevalence and overestimate infection fatality rate because of the healthy volunteer effect. I excluded studies on health-care workers, since this group is at a potentially high exposure risk, which may result in seroprevalence estimates much higher than the general population and thus an improbably low infection fatality rate. Similarly, I also excluded studies on communities (e.g. shelters or religious or other shared-living communities). Studies were eligible regardless of whether they aimed to evaluate seroprevalence in large or small regions, provided that the population of reference in the region was at least 5000 people.

I searched PubMed® (LitCOVID), and medRxiv, bioRxiv and Research Square using the terms “seroprevalence” OR “antibodies” with continuous updates. I made the first search in early May and did monthly updates, with the last update on 9 September 2020. I contacted field experts to retrieve any important studies that may have been missed.

From each study, I extracted information on location, recruitment and sampling strategy, dates of sample collection, sample size, types of antibody measured (immunoglobulin G (IgG), IgM and IgA), the estimated crude seroprevalence (positive samples divided by all samples tested), adjusted seroprevalence and the factors that the authors considered for adjustment.

### Inferred infection fatality rate

If a study did not cover an entire country, I collected information on the population of the relevant location from the paper or recent census data so as to approximate as much as possible the relevant catchment area (e.g. region(s) or county(ies)). Some studies targeted specific age groups (e.g. excluding elderly people and/or excluding children) and some estimated numbers of people infected in the population based on specific age groups. For consistency, I used the entire population (all ages) and, separately, the population 0–70 years to estimate numbers of infected people. I assumed that the seroprevalence would be similar in different age groups, but I also recorded any significant differences in seroprevalence across age strata so as to examine the validity of this assumption.

I calculated the number of infected people by multiplying the relevant population size and the adjusted estimate of seroprevalence. If a study did not give an adjusted seroprevalence estimate, I used the unadjusted seroprevalence instead. When seroprevalence estimates with different adjustments were available, I selected the analysis with largest adjustment. The factors adjusted for included COVID-19 test performance, sampling design, and other factors such as age, sex, clustering effects or socioeconomic factors. I did not adjust for specificity in test performance when positive antibody results were already validated by a different method.

For the number of COVID-19 deaths, I chose the number of deaths accumulated until the date 1 week after the midpoint of the study period (or the date closest to this that had available data) – unless the authors of the study had strong arguments to choose some other time point or approach. The 1-week lag accounts for different delays in developing antibodies versus dying from infection. The number of deaths is an approximation because it is not known when exactly each patient who died was infected. The 1-week cut-off after the study midpoint may underestimate deaths in places where patients are in hospital for a long time before death, and may overestimate deaths in places where patients die soon because of poor or even inappropriate care. Whether or not the health system became overloaded may also affect the number of deaths. Moreover, because of imperfect diagnostic documentation, COVID-19 deaths may have been both overcounted and undercounted in different locations and at different time points. 

I calculated the inferred infection fatality rate by dividing the number of deaths by the number of infected people for the entire population, and separately for people younger than 70 years. I took the proportion of COVID-19 deaths that occurred in people younger than 70 years from situational reports for the respective locations that I retrieved at the time I identified the seroprevalence studies. I also calculated a corrected infection fatality rate to try and account for the fact that only one or two types of antibodies (among IgG, IgM, IgA) might have been used. I corrected seroprevalence upwards (and inferred infection fatality rate downwards) by one tenth of its value if a study did not measure IgM and similarly if IgA was not measured. This correction is reasonable based on some early evidence,^7^ although there is uncertainty about the exact correction factor.

### Data synthesis

The estimates of the infection fatality rate across all locations showed great heterogeneity with *I*^2^ exceeding 99.9%; thus, a meta-analysis would be inappropriate to report across all locations. Quantitative synthesis with meta-analysis across all locations would also be misleading since locations with high COVID-19 seroprevalence would tend to carry more weight than locations with low seroprevalence. Furthermore, locations with more studies (typically those that have attracted more attention because of high death tolls and thus high infection fatality rates) would be represented multiple times in the calculations. In addition, poorly conducted studies with fewer adjustments would get more weight because of spuriously narrower confidence intervals than more rigorous studies with more careful adjustments which allow for more uncertainty. Finally, with a highly skewed distribution of the infection fatality rate and with large between-study heterogeneity, typical random effects models would produce an incorrectly high summary infection fatality rate that approximates the mean of the study-specific estimates (also strongly influenced by high-mortality locations where more studies have been done); for such a skewed distribution, the median is more appropriate.

Therefore, in a first step, I grouped estimates of the infection fatality rate from studies in the same country (or for the United States of America, the same state) together and calculated a single infection fatality rate for that location, weighting the study-specific infection fatality rates by the sample size of each study. This approach avoided inappropriately giving more weight to studies with higher seroprevalence estimates and those with seemingly narrower confidence intervals because of poor or no adjustments, while still giving more weight to larger studies. Then, I used the single summary estimate for each location to calculate the median of the distribution of location-specific infection fatality rate estimates. Finally, I explored whether the location-specific infection fatality rates were associated with the COVID-19 mortality rate in the population (COVID-19 deaths per million people) in each location as of 12 September 2020; this analysis allowed me to assess whether estimates of the infection fatality rate tended to be higher in locations with a higher burden of death from COVID-19.

## Results

### Seroprevalence studies

**Fig. 1:**
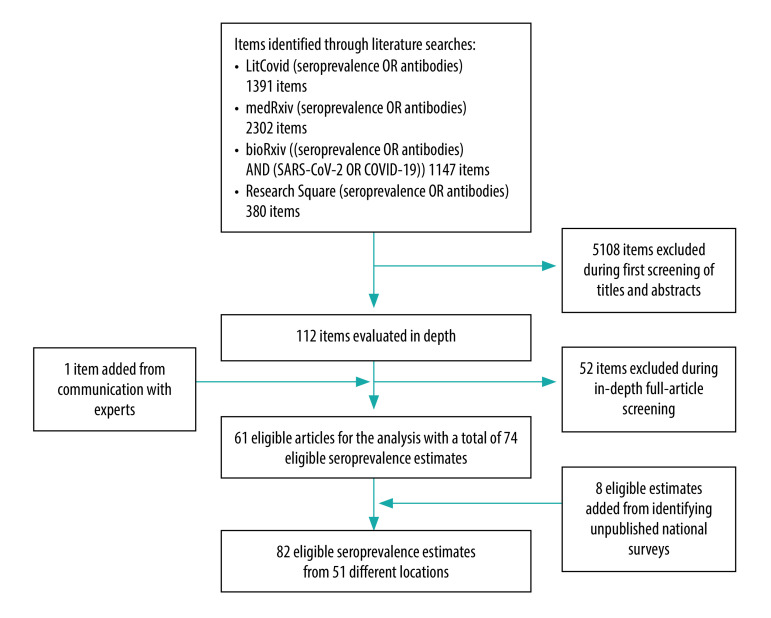
Flowchart for selection of seroprevalence studies on severe acute respiratory syndrome coronavirus 2, 2020

COVID-19: coronavirus disease 2019; SARS-CoV-2: severe acute respiratory syndrome coronavirus 2.

I retrieved 61 studies with 74 eligible estimates published either in the peer-reviewed literature or as preprints as of 9 September 2020.^8^^–^^68^ Furthermore, I considered another eight preliminary national estimates.^69^^–^^76^ This search yielded a total of 82 eligible estimates (Fig. 1).

The studies varied substantially in sampling and recruitment designs (Table 1; available at: http://www.who.int/bulletin/volumes/99/1/20-265892). Of the 61 studies, 24 studies^8^^,^^10^^,^^16^^,^^17^^,^^20^^,^^22^^,^^25^^,^^33^^,^^34^^,^^36^^,^^37^^,^^42^^,^^46^^–^^49^^,^^52^^–^^54^^,^^57^^, ^^61^^,^^63^^,^^65^^,^^68^ explicitly aimed for random sampling from the general population. In principle, random sampling is a stronger design. However, even then, people who cannot be reached (e.g. by email or telephone or even by visiting them at a house location) will not be recruited, and these vulnerable populations are likely to be missed. Moreover, several such studies^8^^,^^10^^,^^16^^,^^37^^,^^42^ focused on geographical locations with high numbers of deaths, higher than other locations in the same city or country, and this emphasis would tend to select eventually for a higher infection fatality rate on average.

**Table 1:** Eligible seroprevalence studies on COVID-19 published or deposited as preprints as of 9 September 2020: dates, sampling and recruitment

Author	Country (location)	Dates	Sampling and recruitment
Figar et al.^47^	Argentina (Barrio Padre Mugica)	10–26 June	Probabilistic sampling of a slum neighbourhood, sampling from people 14 years or older across households
Herzog et al.^38^	Belgium	30 March–5 April and 20–26 April	Residual sera from 10 private diagnostic laboratories in Belgium, with fixed numbers per age group, region and periodical sampling, and stratified by sex
Hallal et al.^25^	Brazil	15–22 May	Sampling from 133 cities (the main city in each region), selecting 25 census tracts with probability proportionate to size in each sentinel city, and 10 households at random in each tract. Aiming for 250 participants per city
Gomes et al.^34^	Brazil (Espirito Santo)	13–15 May	Cross-section of major municipalities with houses as the sampling units
Da Silva et al.^68^	Brazil (Maranhao)	27 July–8 August	Three-stage cluster sampling stratified by four state regions in the state of Maranhao; the estimates took clustering, stratification and non-response into account
Amorim Filho et al.^41^	Brazil (Rio de Janeiro)	14–27 April (eligible: 24–27 April)	Blood donors without flulike symptoms within 30 days of donation; had close contact with suspected or confirmed COVID-19 cases in the 30 days before donation; or had travelled abroad in the past 30 days
Silveira et al.^17^	Brazil (Rio Grande do Sul)	9–11 May (third round, after 11–13 April, and 25–27 April)	Multistage probability sampling in each of nine cities to select 500 households, from which one member was randomly chosen for testing
Tess et al.^42^	Brazil (Sao Paulo)	4–12 May	Randomly selected adults and their cohabitants sampled from six districts of Sao Paulo City with high numbers of cases
Skowronski et al.^50^	Canada (British Columbia)	15–27 May (after baseline in 5–13 March)	Specimens from patients attending one of about 80 diagnostic service centres of the only outpatient laboratory network in the Lower Mainland
Torres et al.^43^	Chile (Vitacura)	4–19 May	Classroom stratified sample of children and all staff in a community placed on quarantine after school outbreak
Chang et al.^55^	China	January–April weekly: 3–23 February (Wuhan); 24 February–15 March (Shenzhen); 10 February–1 March (Shijiazhuang)	38 144 healthy blood donors in Wuhan, Shenzhen and Shijiazhuang who met the criteria for blood donation during the COVID-19 pandemic in China
Wu et al.^14^	China (Wuhan)	3–15 April	People applying for permission to resume work (*n* = 1021) and hospitalized patients (*n* = 381)
Ling et al.^32^	China (Wuhan)	26 March–28 April	Age 16–64 years, going back to work, with no fever, headache or other symptoms of COVID-19
Xu et al.^60^	China (Guangzhou)	23 March–2 April	Healthy blood donors in Guangzhou
Xu et al.^40^	China (several regions)	30 March–10 April	Voluntary participation by public call for haemodialysis patients (*n* = 979 in Jingzhou, Hubei and *n* = 563 in Guangzhou/Foshan, Guangdong) and outpatients in Chongqing (*n* = 993), and community residents in Chengdu, Sichuan (*n* = 9442), and required testing for factory workers in Guangzhou, Guandong (*n* = 442)
Jerkovic et al.^26^	Croatia	23–28 April	DIV Group factory workers in Split and Sibenik-Knin invited for voluntary testing
Erikstrup et al.^12^	Denmark	6 April–3 May	All Danish blood donors aged 17–69 years giving blood. Blood donors are healthy and must comply with strict eligibility criteria; they must self-defer for two weeks if they develop fever with upper respiratory symptoms
Petersen et al.^52^	Denmark (Faroe Islands)	27 April–1 May	1 500 randomly selected residents invited to participate, samples collected from 1075
Fontanet et al.^39^	France (Crepy-en-Valois)	28–30 April	Pupils, their parents and relatives, and staff of primary schools exposed to SARS-CoV-2 in February and March 2020 in a city north of Paris
Fontanet et al.^13^	France (Oise)	30 March–4 April	Pupils, their parents and siblings, as well as teachers and non-teaching staff of a high-school
Streeck et al.^16^	Germany (Gangelt)	30 March–6 April	600 adults with different surnames in Gangelt were randomly selected; all household members were asked to participate in the study
Kraehling et al.^21^	Germany (Frankfurt)	6–14 April	Employees of Infraserv Höchst, a large industrial site operator in Frankfurt am Main. No exclusion criteria
Bogogiannidou et al.^62^	Greece	March and April (April data used)	Leftover blood samples collected from a nationwide laboratory network, including both private and public hospital laboratories (27 laboratories in total)
Merkely et al.^57^	Hungary	1–16 May	Representative sample (*n* = 17 787) of the Hungarian population ≥ 14 years living in private households ( 8 283 810)
Gudbjartsson et al.^58^	Iceland	Several cohorts between April and June^a^	30 576 people in Iceland, including those documented to be infected, those quarantined and people not known to have been exposed
Malani et al.^61^	India (Mumbai)	29 June–19 July	Geographically-spaced community sampling of households, one individual per household was tested in slum and non-slum communities in three wards, one each from the three main zones of Mumbai
Khan et al.^67^	India (Srinagar)	1–15 July	Adults (> 18 years) who visited selected hospitals across the Srinagar District
Shakiba et al.^8^	Islamic Republic of Iran (Guilan)	April (until 21 April)	Population-based cluster random sampling design through telephone call invitation, household-based
Fiore et al.^31^	Italy (Apulia)	1–31 May	Blood donors 18–65 years old free of recent symptoms possibly related to COVID-19, no close contact with confirmed cases, symptom-free in the preceding 14 days, no contact with suspected cases
Doi et al.^11^	Japan (Kobe)	31 March–7 April	Randomly selected patients who visited outpatient clinics and received blood testing for any reason. Patients who visited the emergency department or the designated fever consultation service were excluded
Takita et al.^29^	Japan (Tokyo)	21 April–20 May	Two community clinics in the main railway stations in Tokyo (Navitas Clinic Shinjuku and Tachikawa)
Nawa et al.^48^	Japan (Utsunomiya City)	14 June–5 July	Invitations enclosed with a questionnaire were sent to 2290 people in 1 000 households randomly selected from Utsunomiya City’s basic resident registry; 742 completed the study
Uyoga et al.^44^	Kenya	30 April–16 June (~90% of samples in last 30 days)	Residual blood donor serum samples from donors 16–65 years in four sites (Mombasa, Nairobi, Eldoret and Kisumu)
Snoeck et al.^20^	Luxembourg	16 April–5 May	Representative sample (no details how ensured), 1807 of 2000 contacted provided data, were < 79 years and had serology results
Slot et al.^15^	Netherlands	1–15 April	Blood donors. Donors must be completely healthy, but they may have been ill in the past, provided that they recovered at least 2 weeks before
Westerhuis et al.^64^	Netherlands (Rotterdam)	Early March and early April	Left-over plasma samples from patients of nine age categories in Erasmus Medical Center in Rotterdam: 879 samples in early March and 729 in early April)
Nisar et al.^49^	Pakistan (Karachi)	25 June–11 July (after baseline on 15–25 April)	Cross-sectional household surveys in a low- (district Malir) and high-transmission (district East) area of Karachi with households selected using simple random sampling (Malir) and systematic random sampling (East)
Javed et al.^66^	Pakistan (urban Karachi, Lahore, Multan, Peshawar and Quetta)	Up to 6 July	Adult, working population aged 18–65 years, recruited from dense, urban workplaces including factories, businesses, restaurants, media houses, schools, banks, hospitals (health-care providers), and from families of positive cases in cities in Pakistan
Abu Raddad et al.^51^	Qatar	12 May–12 July (highest seroprevalence on 12–31 May)	Convenience sample of residual blood specimens collected for routine clinical screening or clinical management from 32 970 outpatient and inpatient departments for a variety of health conditions (*n* = 937 in 12–31 May)
Noh et al.^59^	Republic of Korea	25–29 May	Outpatients who visited two hospitals in south-west Seoul which serve six administrative areas
Pollán et al.^36^	Spain	27 April–11 May	35 883 households selected from municipal rolls using two-stage random sampling stratified by province and municipality size, with all residents invited to participate (75.1% of all contacted individuals participated)
Crovetto et al.^30^	Spain (Barcelona)	14 April–5 May	Consecutive pregnant women for first trimester screening or delivery in two hospitals
Stringhini et al.^10^	Switzerland (Geneva)	6 April–9 May (5 consecutive weeks)	Randomly selected previous participants of the Bus Santé study with an email (or telephone contact, if email unavailable); participants were invited to bring all members of their household aged 5 years and older
Emmenegger et al.^28^	Switzerland (Zurich)	Prepandemic until June (patients) and May (blood donors)	Patients at the University Hospital of Zurich and blood donors in Zurich and Lucerne
Ward et al.^65^	United Kingdom (England)	20 June–13 July	Random population sample of 100 000 adults over 18 years
Thompson et al.^18^	United Kingdom (Scotland)	21–23 March	Blood donors. Donors should not have felt unwell in the past 14 days; some other deferrals also applied regarding travel and COVID-19 symptoms
Havers et al.^35^	USA (10 states)	23 March–1 April (Washington, Puget Sound and New York, New York City), 1–8 April (Louisiana), 5–10 April (Florida, south), 13–25 April (Pennsylvania, Philadelphia, metropolitan area), 20–26 April (Missouri), 23–27 April (California, San Francisco Bay Area), 20 April–3 May (Utah), 26 April–3 May (Connecticut), 30 April–12 May (Minnesota, Minneapolis)	Convenience samples using residual sera obtained for routine clinical testing (screening or management) by two commercial laboratory companies
Ng et al.^24^	USA (California, Bay Area)	March	1000 blood donors in diverse Bay Area locations (excluding those with self-reported symptoms or abnormal vital signs)
Sood^22^	USA (California, Los Angeles)	10–14 April	Proprietary database representative of the county. A random sample of these residents was invited, with quotas for enrolment for subgroups based on age, sex, race and ethnicity distribution
Chamie et al.^33^	USA (California, San Francisco)	25–28 April	United States census tract 022 901 population-dense area (58% Latin American) in San Francisco Mission district, expanded to neighbouring blocks on 28 April
Bendavid et al.^19^	USA (California, Santa Clara)	2–3 April	Facebook advertisement with additional targeting by zip code
Biggs et al.^53^	USA (Georgia, DeKalb and Fulton)	28 April–3 May	Two-stage cluster sampling design used to randomly select 30 census blocks in DeKalb County and 30 census blocks in Fulton County, with a target of seven participating households per census block
McLaughlin et al.^46^	USA (Idaho, Blaine County)	4–19 May	Volunteers who registered via a secure web link, using prestratification weighting to the population distribution by age and sex within each zip code
Bryan et al.^9^	USA (Idaho, Boise)	Late April	People from the Boise, Idaho metropolitan area, part of the Crush the Curve initiative
Menachemi et al.^54^	USA (Indiana)	25–29 April	Stratified random sampling among all persons aged ≥ 12 years using Indiana’s 10 public health preparedness districts as sampling strata
Feehan et al.^63^	USA (Louisiana, Baton Rouge)	15–31 July	Representative sample in a method developed by Public Democracy
Feehan et al.^37^	USA (Louisiana, Orleans and Jefferson Parish)	9–15 May	Pool of potential participants reflecting the demographics of the parishes was based on 50 characteristics, then a randomized subset of 150 000 people was selected, and 25 000 were approached with digital apps, and 2640 were recruited
Rosenberg et al.^23^	USA (New York)	19–28 April	Convenience sample of people ≥ 18 years living in New York State, recruited consecutively on entering 99 grocery stores and through an in-store flyer
Meyers et al.^56^	USA (New York)	2–30 March (Columbia University Medical Center, New York City); 13–28 March (CareMount central laboratory)	Discarded clinical samples in Columbia Medical Center, New York City (*n* = 814 in 24 February–30 March, 742 of those in the period 2–30 March) and samples from CareMount central laboratory (960 samples on 13/14 March, 505 samples on 20/21 March, and 376 samples on 27/28 March) from its network of clinics in five counties north of New York City
Reifer et al.^27^	USA (New York, Brooklyn)	Early May	Patients seen in an urgent care facility in Brooklyn
Nesbitt et al.^45^	USA (Rhode Island)	27 April–11 May	Consecutive blood donors

COVID-19: coronavirus disease 19; SARS-CoV-2: severe acute respiratory syndrome coronavirus 2.

^a^ Sample collection time for some sub-cohorts may have exceeded 1 month, but more than half of the cases were already documented by polymerase chain reaction testing before any antibody testing and the last death occurred on 20 April.

Note: Some studies included additional data sets that did not fulfil the eligibility criteria (e.g. had sample size < 500 or were health-care workers) and they are not presented here.

Eleven studies assessed blood donors,^12^^,^^15^^,^^18^^,^^24^^,^^28^^,^^31^^,^^41^^,^^44^^,^^45^^,^^55^^,^^60^ which might underestimate COVID-19 seroprevalence in the general population. For example, 200 blood donors in Oise, France showed 3.00% seroprevalence, while the seroprevalence was 25.87% (171/661) in pupils, siblings, parents, teachers and staff at a high school with a cluster of cases in the same area; the true population seroprevalence may be between these two values.^13^

For other studies, healthy volunteer bias^19^ may underestimate seroprevalence, attracting people with symptoms^26^ may overestimate seroprevalence, and studies of employees,^14^^,^^21^^,^^25^^,^^32^^,^^66^ grocery store clients^23^ or patient cohorts^11^^,^^14^^,^^27^^–^^30^^,^^36^^,^^38^^,^^40^^,^^50^^,^^51^^,^^56^^,^^59^^,^^62^^,^^64^^,^^67^ risk sampling bias in an unpredictable direction.

All the studies tested for IgG antibodies but only about half also assessed IgM and few assessed IgA. Only seven studies assessed all three types of antibodies and/or used pan-Ig antibodies. The ratio of people sampled versus the total population of the region was more than 1:1000 in 20 studies (Table 2; available at: http://www.who.int/bulletin/volumes/99/1/20-265892).

**Table 2:** Sample size, types of antibodies assessed and population size in the studies included to assess COVID-19 infection fatality rate, 2020

Country (location)	Sample size^a^, no.	Antibody	Population,^b,c.d^ no.	% of population < 70 years^c^
**Argentina (Barrio Padre Mugica)**^47^	873	IgG	49 983	99
**Belgium**^38^	3 391 (20–26 April)	IgG	11 589 623	86
**Brazil (133 cities)**^25^	24 995	IgG and IgM	74 656 499	94 (Brazil)
**Brazil (Espirito Santo)**^34^	4 608	IgG and IgM	4 018 650	94 (Brazil)
**Brazil (Maranhao)**^68^	3 156	IgG and IgM	7 114 598	92
**Brazil (Rio de Janeiro), blood donors**^41^	669 (24–27 April)	IgG and IgM	17 264 943	94 (Brazil)
**Brazil (Rio Grande do Sul)**^17^	4 500	IgG	11 377 239	91
**Brazil (Sao Paulo)**^42^	517	IgG and IgM	298 240 (6 districts)	94 (Brazil)
**Canada (British Columbia)**^50^	885	IgG, IgM and IgA	5 071 000	94
**Chile (Vitacura)**^43^	1 244	IgG and IgM	85 000	92 (Chile)
**China, blood donors**^55^				
Wuhan	930 (3–23 February)	IgG and IgM	11 210 000	93 (China)
Shenzhen	3 507 (24 February–15 March)	IgG and IgM	13 030 000	93 (China)
Shijiazhuang	6 455 (10 February–1 March)	IgG and IgM	11 030 000	93 (China)
**China (Wuhan)**^14^	1 401	IgG and IgM	11 080 000	93 (China)
**China (Wuhan)**^32^	1 196 (4–8 April)	IgG and IgM	11 080 000	93 (China)
**China (Guangzhou), blood donors**^60^	2 199	IgG, IgM and IgA	115 210 000 (Guangdong)	93 (China)
**China (several regions)**^40^				
Hubei (not Wuhan)	979	IgG and IgM	48 058 000	93 (China)
Chongqing	993	IgG and IgM	31 243 200	93 (China)
Sichuan	9 442	IgG and IgM	83 750 000	93 (China)
Guangdong	1 005	IgG and IgM	115 210 000	93 (China)
**Croatia**^26^	1 494	IgG and IgM	4 076 000	86
**Denmark blood donors**^12^	20 640	IgG and IgM	5 771 876	86
**Denmark (Faroe Islands)**^52^	1 075	IgG and IgM	52 428	88
**France (Crepy-en-Valois)**^39^	1 340	IgG	5 978 000 (Hauts-de-France)	89
**France (Oise)**^13^	661	IgG	5 978 000 (Hauts-de-France)	89
**Germany (Gangelt)**^16^	919	IgG and IgA	12 597	86
**Germany (Frankfurt)**^21^	1 000	IgG	2 681 000^e^	84 (Germany)
**Greece**^62^	6 586 (4 511 in April)	IgG	10 412 967	84
**Hungary**^57^	10 504	IgG (also had RT-PCR)	9 657 451	88
**Iceland**^58^	30 576	Pan-Ig	366 854	90
**India (Mumbai)**^61^	6 904 (4 202 in slums, 2 702 not in slums)	IgG	1 414 917 (705 523 in slums, 709 394 in non-slums) in the 3 ward areas	98
**India (Srinagar)**^67^	2 906	IgG	1 500 000	97
**Islamic Republic of Iran (Guilan)**^8^	551	IgG and IgM	2 354 848	95
**Italy (Apulia), blood donors**^31^	909	IgG and IgM	4 029 000	84
**Japan (Kobe)**^11^	1 000	IgG	1 518 870	79 (Japan)
**Japan (Tokyo)**^29^	1 071	IgG	13 902 077	79 (Japan)
**Japan (Utsunomiya City)**^48^	742	IgG	518 610	79 (Japan)
**Kenya, blood donors**^44^	3 098	IgG	47 564 296	99
**Luxembourg**^20^	1 807	IgG and IgA^f^	615 729	90
**Netherlands blood donors**^15^	7 361	IgG, IgM and IgA	17 097 123	86
**Netherlands (Rotterdam)**^64^	729 (early April)	IgG	17 097 123 (Netherlands)	86
**Pakistan (Karachi)**^49^	1 004	IgG and IgM	16 700 000	98 (Pakistan)
**Pakistan (urban)**^66^	24 210	IgG and IgM	79 000 000 (urban)	98
**Qatar**^51^	937	IgG	2 800 000	99
**Republic of Korea**^59^	1 500	IgG	2 667 341	90 (Republic of Korea)
**Spain**^36^	61 075	IgG	46 940 000	85
**Spain (Barcelona)**^30^	874	IgG, IgM and IgA	7 566 000 (Catalonia)	86
**Switzerland (Geneva)**^10^	577 (20–27 April)	IgG	500 000	88
**Switzerland (Zurich)**^28^	1 644 patients (1–15 April)	IgG	1 520 968 (Zurich canton)	88
**Switzerland (Zurich and Lucerne)**^28^	1 640 blood donors (May)	IgG	1 930 525 (Zurich and Lucerne)	88
**United Kingdom (England)**^65^	109 076	IgG	56 287 000	86
**United Kingdom (Scotland), blood donors**^18^	500	IgG	5 400 000	88
**USA (10 states)**^35^				
Washington, Puget Sound	3 264	Pan-Ig	4 273 548	90 (Washington)
Utah	1 132	Pan-Ig	3 282 120	92
New York, New York City	2 482	Pan-Ig	9 260 870	89
Missouri	1 882	Pan-Ig	6 110 800	88
Florida, south	1 742	Pan-Ig	6 345 345	86 (Florida)
Connecticut	1 431	Pan-Ig	3 562 989	88
Louisiana	1 184	Pan-Ig	4 644 049	92
California, San Francisco Bay	1 224	Pan-Ig	2 173 082	90
Pennsylvania, Philadelphia	824	Pan-Ig	4 910 139	90
Minnesota, Minneapolis	860	Pan-Ig	3 857 479	90
**USA (California, Bay Area)**^24^	1 000	IgG	7 753 000	90
**USA (California, Los Angeles)**^22^	863	IgG and IgM	7 892 000	92
**USA (California, San Francisco)**^33^	3 953	IgG and RT-PCR	5 174 (in census tract 022 901)	95
**USA (California, Santa Clara)**^19^	3 300	IgG and IgM	1 928 000	90
**USA (Idaho, Boise)**^9^	4 856	IgG	481 587 (Ada County)	92
**USA (Georgia, DeKalb and Fulton Counties)**^53^	696	Total Ig	1 806 672	88 (Georgia)
**USA (Idaho, Blaine County)**^46^	917	IgG	23 089	92
**USA (Indiana)**^54^	3 629	IgG and RT-PCR	6 730 000	89
**USA (Louisiana, Baton Rouge)**^63^	138	IgG	699 200 (East Baton Rouge, West Baton Rouge, Ascension, Livingston)	92 (Louisiana)
**USA (Louisiana, Orleans and Jefferson Parish)**^37^	2 640	IgG	825 057	92 (Louisiana)
**USA (New York)**^23^	15 101	IgG	19 450 000	90
**USA, New York**^56^				
Columbia University Medical Center, New York City	742 (2–30 March)	IgG and IgM	9 260 870	89
CareMount central laboratory, five New York state counties	1 841	IgG and IgM	10 189 130 (New York state excluding New York City)	89
**USA (New York, Brooklyn)**^27^	11 092	IgG	2 559 903	91
**USA (Rhode Island), blood donors**^45^	1 996	IgG and IgM	1 059 000	88

COVID-19: coronavirus disease 19; Ig: immunoglobin; RT-PCR: real-time polymerase chain reaction.

^a^ Dates in brackets are the specific dates used when seroprevalence was evaluated at multiple consecutive time points or settings.

^b^ Some studies focused on age-restricted populations of the specific location under study, for example: people 17–70 years in the Denmark blood donor study (*n* = 3 800 000); people 18–79 years in the Luxembourg study (*n* = 483 000); people < 70 years in the Netherlands blood donor study (*n* = 13 745 768); people ≥ 18 years in the New York state study (*n* = 15 280 000); people > 19 years in the Utah population of the 10-state United States of America study (*n* = 2 173 082); people ≥ 18 years in Blaine County, Idaho (*n*  = 17 611); people 15–64 years in the Kenya blood donor study (*n* = 27 150 165); people > 14 years living in private premises in Hungary (*n* = 8,283,810); people > 18 years (*n*  = 551 185) in Baton Rouge, Louisiana; people 18–65 years working in urban locations in Pakistan (*n* = 22 100 000); and people > 18 years in Srinagar District, India (*n* = 1 020 000). In this table and subsequent analyses, the entire population in the location is considered for consistency across studies.

^c^ Information in parentheses specifies the population.

^d^ When the population of the relevant location was not given in a specific study, information on recent estimates of the pertinent population was obtained by standard online sources such as: populationpyramid.net, worldpopulationreview.com, worldometers.info/coronavirus, and Wikipedia.

^e^ Participants were recruited from a large number of districts, but most districts had very few participants; here I included the population of the nine districts with > 1:10 000 sampling ratio (846/1000 participants came from these nine districts).

^f^ Considered positive if both IgG and IgA were positive; in the other studies, detection of any antibody was considered positive.

### Seroprevalence estimates

Seroprevalence for the infection ranged from 0.02% to 53.40% (58.40% in the slum sub-population in Mumbai; Table 3). Studies varied considerably depending on whether or not they tried to adjust their estimates for test performance, sampling (to get closer to a more representative sample), clustering (e.g. when including household members) and other factors. The adjusted seroprevalence occasionally differed substantially from the unadjusted value. In studies that used samples from multiple locations, between-location heterogeneity was seen (e.g. 0.00–25.00% across 133 Brazilian cities).^25^

**Table 3:** Estimated prevalence of COVID-19 and estimated number of people infected, 2020

Country (location)	Seroprevalence, %			Estimated no. of people infected
	Crude	Adjusted		
		Value	Adjustments	
**Argentina (Barrio Padre Mugica)**^47^	ND	53.4	Age, sex, household, non-response	26 691
**Belgium**^38^	5.7	6.0	Sampling, age, sex, province	695 377
**Brazil (133 cities)**^25^	1.39	1.62 overall (0–25.0 across the 133 cities)	Test, design	1 209 435^a^
**Brazil (Espirito Santo)**^34^	2.1	ND	NA	84 391
**Brazil (Maranhao)**^68^	37	40.4	Clustering, stratification, non-response	2 877 454
**Brazil (Rio de Janeiro), blood donors**^41^	6	4.7	Age, sex, test	811 452
**Brazil (Rio Grande do Sul)**^17^	0.222	0.222^b^	Sampling	25 283
**Brazil (Sao Paulo)**^42^	5.2	4.7	Sampling design	14 017
**Canada (British Columbia)**^50^	0.45	0.55	Age	27 890
**Chile (Vitacura)**^43^	11.2	ND	NA	9 500
**China, blood donors**^55^				
Wuhan	3.87	ND	NA	433 827
Shenzhen	0.06	ND	NA	7 818
Shijiazhuang	0.02	ND	NA	2 206
**China (Wuhan)**^14^	10	ND	NA	1 108 000
**China (Wuhan)**^32^	8.36	ND	NA	926 288
Entire period	3.53	2.80	Age, sex, test	–
**China (Guangzhou), blood donors**^60^	0.09	ND	NA	104 783
**China (several regions)**^40^				
Hubei (not Wuhan)	3.6	ND	NA	1 718 110
Chongqing	3.8	ND	NA	11 956 109
Sichuan	0.6	ND	NA	487 847
Guangdong	2.2	ND	NA	2 522 010
**Croatia**^26^	1.27^c^	ND	NA	51 765
**Denmark, blood donors**^12^	2	1.9	Test	109 665
**Denmark (Faroe Islands)**^52^	0.6	0.7	Test	365
**France (Crepy-en-Valois)**^39^	10.4	ND	NA	620 105
**France (Oise)**^13^	25.9	ND	NA	1 548 000
**Germany (Gangelt)**^16^	15	20.0	Test, cluster, symptoms	2 519
**Germany (Frankfurt)**^21^	0.6	ND	NA	16 086
**Greece**^62^	0.42 (April)	0.49^d^	Age, sex, region	51 023
**Hungary**^57^	0.67	0.68	Design, age, sex, district	65 671
**Iceland**^58^	2.3 (quarantined), 0.3 (unknown exposure)	0.9	Including those positive by RT-PCR	3 177
**India (Mumbai)**^61^				534 750
Slum areas	54.1	58.4	Test, age, sex	–
Non-slum areas	16.1	17.3	Test, age, sex	–
**India (Srinagar)**^67^	3.8	3.6	Age, sex	54 000
**Islamic Republic of Iran (Guilan)**^8^	22	33.0	Test, sampling	770 000
**Italy (Apulia), blood donors**^31^	0.99	ND	NA	39 887
**Japan (Kobe)**^11^	3.3	2.7	Age, sex	40 999
**Japan (Tokyo)**^29^	3.83	ND	NA	532 450
**Japan (Utsunomiya City)**^48^	0.4	1.23	Age, sex, distance to clinic, district, cohabitants	6 378
**Kenya, blood donors**^44^	5.6	5.2	Age, sex, region, test	2 783 453
**Luxembourg**^20^	1.9	2.1	Age, sex, district	12 684
**Netherlands, blood donors**^15^	2.7	ND	NA	461 622
**Netherlands (Rotterdam)**^64^	3	ND	NA	512 910
**Pakistan (Karachi)**^49^	16.3	11.9	Age, sex	1 987 300
East	20.0	15.1	Age, sex	–
Malir	12.7	8.7	Age, sex	–
**Pakistan (urban)**^66^	17.5	ND	NA	13 825 000
**Qatar**^51^	30.4	ND	NA	851 200
Entire period	24.0	ND	NA	–
**Republic of Korea**^59^	0.07	ND	NA	1 867
**Spain**^36^	ND	5.0^e^	Sampling, age, sex, income	2 347 000
**Spain (Barcelona)**^30^	14.3	ND	NA	1 081 938
**Switzerland (Geneva)**^10^	10.6	10.9	Test, age, sex	54 500
**Switzerland **^28^				
Zurich^f^	Unclear	1.3	Multivariate Gaussian conditioning	19 773
Zurich and Lucerne^g^	Unclear	1.6	Multivariate Gaussian conditioning	30 888
**United Kingdom (England)**^65^	5.6	6.0	Test, sampling	3 360 000
**United Kingdom (Scotland) blood donors**^18^	1.2	ND	NA	64 800
**USA (10 states)**^35^				
Washington, Puget Sound	1.3	1.1	Age, sex, test	48 291
Utah	2.4	2.2	Age, sex, test	71 550
New York, New York City	5.7	6.9	Age, sex, test	641 778
Missouri	2.9	2.7	Age, sex, test	161 936
Florida, south	2.2	1.9	Age, sex, test	117 389
Connecticut	4.9	4.9	Age, sex, test	176 012
Louisiana	ND	5.8	Age, sex, test	267 033
California, San Francisco Bay	ND	1	Age, sex, test	64 626
Pennsylvania, Philadelphia	ND	3.2	Age, sex, test	156 633
Minnesota, Minneapolis	ND	2.4	Age, sex, test	90 651
**USA (California, Bay Area)** **blood donors**^24^	0.4	0.1	Test and confirmation	7 753
**USA (California, Los Angeles)**^22^	4.06	4.65	Test, sex, race and ethnicity, income	367 000
**USA (California, San Francisco), in census tract 022 901**^33^	4.3	6.1	Age, sex, race and ethnicity, test	316
**USA (California, Santa Clara)**^19^	1.5	2.6	Test, sampling, cluster	51 000
**USA (Idaho, Boise)**^9^	1.79	ND	NA	8620
**USA (Georgia, DeKalb and Fulton counties)**^53^	2.7	2.5	Age, sex, race and ethnicity	45 167
**USA (Idaho, Blaine County)**^46^	22.4	23.4	Test, age, sex, household	5 403
**USA (Indiana)**^54^	2.3 (IgG and RT-PCR)^h^	2.8	Age, race, Hispanic ethnicity	187 802
**USA (Louisiana, Baton Rouge)**^63^	6	6.6	Census, race, parish, including RT-PCR positives	46 147
**USA (Louisiana, Orleans and Jefferson Parish)**^37^	6.9 (IgG and RT-PCR)^h^	6.9 for IgG	Census weighting, demographics	56 578
**USA (New York)**^23^	12.5	14.0	Test, sex, age race and ethnicity, region	2 723 000
**USA, New York**^56^				
Columbia University Medical Center, New York City	5	ND	NA	463 044
CareMount central laboratory, five New York state counties	1.8	ND	NA	183 404
**USA (New York, Brooklyn)**^27^	47	ND	NA	1 203 154
**USA (Rhode Island), blood donors**^45^	3.9	ND	NA	41 384

COVID-19: coronavirus disease 2019; NA: not applicable; ND: no data available; RT-PCR: real-time polymerase chain reaction; test: test performance.

^a^ The authors calculated 760 000 to be infected in the 90 cities that had 200–250 samples tested, but many of the other 43 cities with < 200 samples may be equally or even better represented since they tended to be smaller than the 90 cities (mean population 356 213 versus 659 326).

^b^ An estimate is also provided adjusting for test performance, but the assumed specificity of 99.0% seems inappropriately low, since as part of the validation process the authors found that several of the test-positive individuals had household members who were also infected, thus the estimated specificity was deemed by the authors to be at least 99.95%.

^c^ 1.20% in workers in Split without mobility restrictions, 3.37% in workers in Knin without mobility restrictions, 1.57% for all workers without mobility restrictions; Split and Knin tended to have somewhat higher death rates than nationwide Croatia, but residence of workers is not given, so the entire population of the country is used in the calculations.

^d^ An estimate is also provided adjusting for test performance resulting in adjusted seroprevalence of 0.23%, but this seems inappropriately low, since the authors report that all positive results were further validated by ELISA (enzyme-linked immunosorbent assay).

^e^ 5.0% with point of care test, 4.6% with immunoassay, 3.7% with both tests positive, 6.2% with at least one test positive.

^f^ Patients during 1–15 April.

^g^ Blood donors in May.

^h^ The study counts in prevalence also those who were currently/recently infected as determined by a positive RT-PCR.

Notes: Of the studies where seroprevalence was evaluated at multiple consecutive time points, the seroprevalence estimate was the highest in the most recent time interval with few exceptions, for example: in the Switzerland (Geneva) study,^10^ the highest value was seen 2 weeks before the last time interval; in the Switzerland (Zurich) study,^28^ the highest value was seen in the period 1–15 April for patients at the university hospital and in May for blood donors; and in the China (Wuhan) study,^32^ the highest value was seen about 3 weeks before the last time interval.

### Inferred infection fatality rate

Inferred infection fatality rate estimates varied from 0.00% to 1.63% (Table 4). Corrected values also varied considerably (0.00–1.54%). 

**Table 4:** Deaths from COVID-19 and inferred infection fatality rates, overall and in people younger than 70 years, by location, 2020

Location	No. of site-specific cumulative deaths from COVID-19 (to date)^a^	Inferred infection fatality rate, % (corrected)	% of site-specific cumulative deaths from COVID-19 in people < 70 years^a^	Infection fatality rate in people < 70 years, % (corrected)
**Argentina (Barrio Padre Mugica)**^47^	44 (1 July)	0.16 (0.13)	~70	0.11 (0.09)
**Belgium**^38^	7594 (30 April)	1.09 (0.87)	10	0.13 (0.10)
**Brazil (133 cities)**^25^	–^b^	Median 0.30 (0.27)	31 (< 60 years)	0.10 (0.09)
**Brazil (Espirito Santo)**^34^	363 (21 May)	0.43 (0.39)	31 (Brazil, < 60 years)	0.14 (0.13)
**Brazil (Maranhao)**^68^	4272 (8 August)	0.15 (0.14)	23	0.04 (0.03)
**Brazil (Rio de Janeiro), blood donors**^41^	1019 (3 May)	0.12 (0.11)	31 (Brazil, < 60 years)	0.04 (0.04)
**Brazil (Rio Grande do Sul)**^17^	124 (14 May)	0.49 (0.39)	31 (Brazil, < 60 years)	0.19 (0.15)
**Brazil (Sao Paulo)**^42^	NA^c^ (15 May)	Unknown, but likely > 0.4	31 (Brazil, < 60 years)	Unknown, but likely > 0.1
**Canada (British Columbia)**^50^	164 (28 May)	0.59 (0.59)	13	0.08 (0.08)
**Chile (Vitacura)**^43^	NA^c^ (18 May)	Unknown, but likely < 0.2	36 (Chile)	Unknown, but likely < 0.1
**China, blood donors**^55^				
Wuhan	1935 (20 February)	0.45 (0.41)	50	0.24 (0.22)
Shenzhen	1 (5 March)	0.01 (0.01)	About 50 (if similar to Wuhan)	0.01 (0.01)
Shijiazhuang	1 (27 February)	0.05 (0.04)	About 50 (if similar to Wuhan)	0.03 (0.02)
**China (Wuhan)**^14^	3869 (2 May)	0.35 (0.31)	50	0.19 (0.15)
**China (Wuhan)**^32^	3869 (13 April)	0.42 (0.38)	50	0.23 (0.21)
**China (Guangzhou), blood donors**^60^	8 (5 April)	0.00 (0.00)	About 50 (if similar to Wuhan)	0.00 (0.00)
**China (several regions)**^40^				
Hubei (not Wuhan)	643 (12 April)	0.04 (0.03)	About 50 (if similar to Wuhan)	0.02 (0.02)
Chongqing	6 (12 April)	0.00 (0.00)	About 50 (if similar to Wuhan)	0.00 (0.00)
Guangdong	8 (12 April)	0.00 (0.00)	About 50 (if similar to Wuhan)	0.00 (0.00)
Sichuan	3 (12 April)	0.00 (0.00)	About 50 (if similar to Wuhan)	0.00 (0.00)
**Croatia**^26^	79 (3 May)	0.15 (0.14)	13	0.02 (0.02)
**Denmark, blood donors**^12^	370 (21 April)	0.34 (0.27)	12	0.05 (0.04)
**Faroe Islands**^52^	0 (5 May)	0.00 (0.00)	0	0.00 (0.00)
**France (Crepy-en-Valois)**^39^	2325 (5 May)^d^	0.37 (0.30)	7 (France, < 65 years)	0.04 (0.03)
**France (Oise)**^13^	932 (7 April)^d^	0.06 (0.05)	7 (France, < 65 years)	0.01 (0.01)
**Germany (Gangelt)**^16^	7 (15 April)	0.28 (0.25)	0	0.00 (0.00)
**Germany (Frankfurt)**^21^	42^e^ (17 April)	0.26 (0.21)	14 (Germany)	0.04 (0.03)
**Greece**^62^	121 (22 April)	0.24 (0.19)	30	0.09 (0.07)
**Hungary**^57^	442 (15 May)	0.67 (0.54)	No data	No data
**Iceland**^58^	10 (1 June)	0.30 (0.30)	30	0.10 (0.10)
**India (Mumbai)**^61^	495 (13–20 July)	0.09 (0.07)	50 (< 60 years, India)	0.04 (0.03)
**India (Srinagar)**^67^	35 (15 July)^f^	0.06 (0.05)	50 (< 60 years, India)	0.03 (0.03)
**Islamic Republic of Iran (Guilan)**^8^	617 (23 April)	0.08 (0.07)	No data	No data
**Italy (Apulia), blood donors**^31^	530 (22 May)	1.33 (1.20)	15 (Italy)	0.24 (0.22)
**Japan (Kobe)**^11^	10 (mid-April)	0.02 (0.02)	21 (Japan)	0.01 (0.01)
**Japan (Tokyo)**^29^	189 (11 May)	0.04 (0.03)	21 (Japan)	0.01 (0.01)
**Japan (Utsunomiya City)**^48^	0 (14 June)	0.00 (0.00)	0	0.00 (0.00)
**Kenya, blood donors**^44^	64 (31 May)	0.00 (0.00)	58 (< 60 years)	0.00 (0.00)
**Luxembourg**^20^	92 (2 May)	0.73 (0.58)	9	0.07 (0.06)
**Netherlands, blood donors**^15^	3134 (15 April)	0.68 (0.68)	11	0.09 (0.09)
**Netherlands (Rotterdam)**^64^	3134 (15 April)	0.65 (0.52)	11	0.08 (0.06)
**Pakistan (Karachi)**^49^	~1500 (9 July)^g^	0.08 (0.07)	~70	0.06 (0.05)
**Pakistan (urban)**^66^	5266 (13 July)^h^	0.04 (0.04)	~70	0.03 (0.03)
**Qatar**^51^	93 (19 June)	0.01 (0.01)	74	0.01 (0.01)
**Republic of Korea**^59^	2 (3 June)^i^	0.10 (0.09)	0	0.00 (0.00)
**Spain**^36^	26 920 (11 May)	1.15 (0.92)	13	0.18 (0.14)
**Spain (Barcelona)**^30^	5137 (2 May)	0.48 (0.48)	13 (Spain)	0.07 (0.07)
**Switzerland (Geneva)**^10^	243 (30 April)	0.45 (0.36)	8	0.04 (0.03)
**Switzerland (Zurich)**^28^	107 (15 April, Zurich), 147 (22 May, Zurich and Lucerne)	0.51 (0.41)	8 (Switzerland)	0.05 (0.04)
**England**^65^	38 854 (9 July)	1.16 (0.93)	20	0.27 (0.22)
**Scotland, blood donors**^18^	47 (1 April)	0.07 (0.06)	9 (< 65 years)	0.01 (0.01)
**USA (10 states)**^35^				
Washington, Puget Sound	207 (4 April)	0.43 (0.43)	10 (state, < 60 years)	0.05 (0.05)
Utah	58 (4 May)	0.08 (0.08)	28 (< 65 years)	0.03 (0.03)
New York	4146 (4 April)	0.65 (0.65)	34 (state)	0.25 (0.25)
Missouri	329 (30 April)	0.20 (0.20)	23	0.05 (0.05)
Florida, south	295 (15 April)	0.25 (0.25)	28 (state)	0.08 (0.08)
Connecticut	2718 (6 May)	1.54 (1.54)	18	0.31 (0.31)
Louisiana	806 (11 April)	0.30 (0.30)	32	0.10 (0.10)
California, San Francisco Bay	321 (1 May)	0.50 (0.50)	25	0.14 (0.14)
Pennsylvania, Philadelphia	697 (26 April)	0.45 (0.45)	21 (state)	0.10 (0.10)
Minnesota, Minneapolis	436 (13 May)	0.48 (0.48)	20 (state)	0.10 (0.10)
**USA (California, Bay Area)**^24^	12 (22 March)	0.15 (0.12)	25	0.04 (0.03)
**USA (California, Los Angeles)**^22^	724 (19 April)	0.20 (0.18)	24 (< 65 years)	0.06 (0.05)
**USA (California, San Francisco)**^33^	0 (4 May)	0.00 (0.00)	0	0.00 (0.00)
**USA (California, Santa Clara)**^19^	94 (22 April)	0.18 (0.17)	35	0.07 (0.06)
**USA (Idaho, Boise)**^9^	14 (24 April)	0.16 (0.13)	14 (Idaho)	0.02 (0.02)
**USA (Georgia)**^53^	198 (7 May)	0.44 (0.44)	30	0.15 (0.15)
**USA (Idaho, Blaine County)**^46^	5 (19 May)	0.10 (0.08)	14 (Idaho)	0.02 (0.01)
**USA (Indiana)**^54^	1099 (30 April)	0.58 (0.46)	24	0.16 (0.13)
**USA (Louisiana, Baton Rouge)**^63^	420 (30 July)	0.91 (0.73)	32 (Louisiana)	0.32 (0.25)
**USA (Louisiana, Orleans and Jefferson Parish)**^37^	925 (16 May)	1.63 (1.31)	32	0.57 (0.46)
**USA (New York)**^23^	18 610 (30 April)^j^	0.68 (0.54)^j^	34	0.26 (0.23)
**USA (New York Columbia University Medical Center, New York City and CareMount central laboratory, five New York state counties)**^56^	965 (28 March, New York state)	0.15 (0.14)	34	0.06 (0.05)
**USA (New York, Brooklyn)**^27^	4894 (19 May)^j^	0.41 (0.33)^j^	34 (New York state)	0.15 (0.14)
**USA (Rhode Island), blood donors**^45^	430 (11 May)	1.04 (0.83)	17	0.20 (0.16)

COVID-19: coronavirus disease 2019; NA: not available.

^a^ Whenever the number or proportion of COVID-19 deaths at age < 70 years was not provided in the paper, I retrieved the proportion of these deaths from situation reports of the relevant location. If I could not find this information for the specific location, I used a larger geographic area. For Brazil, the closest information that I found was from a news report.^77^ For Croatia, I retrieved data on age for 45/103 deaths through Wikipedia.^78^ Geographical location in parentheses specifies the population

^b^ Data are provided by the authors for deaths per 100 000 population in each city along with inferred infection fatality rate in each city, with wide differences across cities; the infection fatality rate shown here is the median across the 36 cities with 200–250 samples and at least one positive sample (the interquartile range for the uncorrected infection fatality rate is 0.20–0.60% and across all cities is 0–2.4%, but with very wide uncertainty in each city). A higher infection fatality rate is alluded to in the preprint, but the preprint also shows a scatter diagram for survey-based seroprevalence versus reported deaths per population with a regression slope that agrees with an infection fatality rate of 0.3%.

^c^ Information on deaths was not available for the specific locations. In the Sao Paulo study, the authors selected six districts of Sao Paulo most affected by COVID-19; they do not name the districts and the number of deaths as of mid-May is not available, but using data for death rates across all Sao Paulo would give an infection fatality rate of > 0.4% overall. In the Vitacura study, similarly one can infer from the wider Santiago metropolitan area that the infection fatality rate in the Vitacura area would probably be < 0.2% overall.

^d^ For France, government situation reports provide the number of deaths per region only for in-hospital deaths; therefore, I multiplied the number of in-hospital deaths by a factor equal to: total number of deaths/in-hospital deaths for all of France.

^e^ Estimated from number of deaths in Hesse province on 17 April × proportion of deaths in the nine districts with key enrolment (enrolment ratio > 1:10 000) in the study among all deaths in Hesse province.

^f^ I calculated the approximate number of deaths assuming the same case fatality ratio in the Srinagar district as in the Jammu and Kashmir state where it is located.

^g^ For Karachi, it is assumed that about 30% of COVID-19 deaths in Pakistan are in Karachi (since about 30% of the cases are there).

^h^ The number of deaths across all Pakistan; I assumed that this number is a good approximation of deaths in urban areas (most deaths occur in urban areas and there is some potential underreporting).

^i^ I calculated the approximate number of deaths from the number of cases in the study areas in south-western Seoul, assuming a similar case fatality as in Seoul overall.

^j^ Confirmed COVID-19 deaths; inclusion of probable COVID-19 deaths would increase the infection fatality rate estimates by about a quarter.

Note: Cumulative deaths are sourced from the specific study or from situation report on the same location unless otherwise stated.

**Fig. 2:**
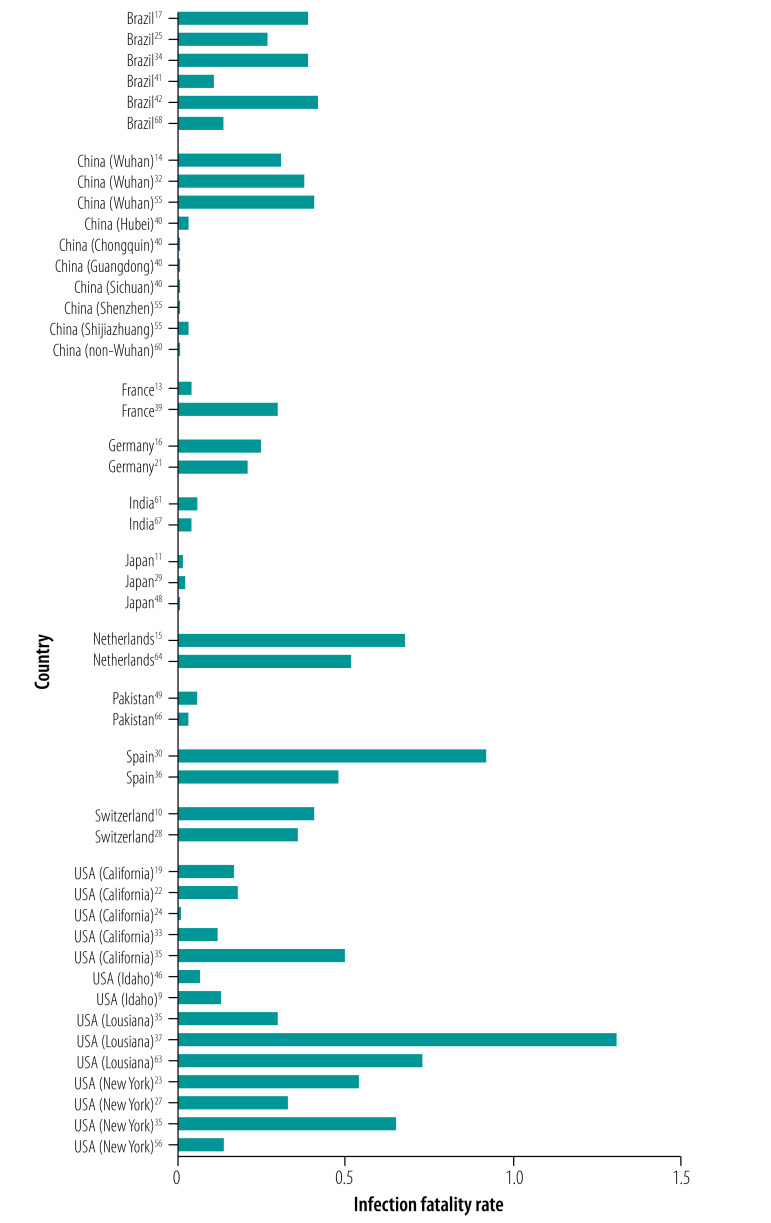
Estimates of infection fatality rates for COVID-19 in locations that had two or more estimates, 2020

For 15 locations, more than one estimate of the infection fatality rate was available and thus I could compare the infection fatality rate from different studies evaluating the same location. The estimates of infection fatality rate tended to be more homogeneous within each location, while they differed markedly across locations (Fig. 2). Within the same location, infection fatality rate estimates tend to have only small differences, even though it is possible that different areas within the same location may also have real differences in infection fatality rate. France is one exception where differences are large, but both estimates come from population studies of outbreaks from schools and thus may not provide good estimates of population seroprevalence and may lead to an underestimated infection fatality rate.

COVID-19: coronavirus disease 2019.

Notes: Locations are defined at the level of countries, except for the United States of America where they are defined at the level of states and China is separated into Wuhan and non-Wuhan areas. Corrected infection fatality rate estimates are shown (correcting for what types of antibodies were assayed).

I used summary estimates weighted for sample size to generate a single estimate for each location. Data were available for 51 different locations (including the inferred infection fatality rates from the eight preliminary additional national estimates in Table 5).

**Table 5:** Infection fatality rates for COVID-19 inferred from preliminary nationwide seroprevalence data, 2020

Country	Sample size	Date	Reported seroprevalence (%)	Population, no.	Deaths, no. (date)	Inferred infection fatality rate (corrected), %
Afghanistan^75^	9 500 (NR)	NR	31.5	39 021 453	1 300 (8 May)	0.01 (0.01)
Czechia^71^	26 549 (IgG)	23 April–1 May	0.4	10 710 000	252 (4 May)	0.59 (0.47)
Finland^69^	674 (IgG)	20–26 April^a^	2.52	5 541 000	211 (30 April)	0.15 (0.12)
Georgia^76^	1 068 (NR)	18–27 May	1	3 988 264	12 (30 May)	0.03 (0.03)^b^
Israel^72^	1 709 (NR)	May	2–3	9 198 000	299 (10 June)	0.13 (0.10)^c^
Russian Federation^74^	650 000 (NR)	NR	14	145 941 776	5 859 (7 June)	0.03 (0.03)
Slovenia^73^	1 368 (NR)	April	3.1	2 079 000	92 (1 May)	0.14 (0.11)
Sweden^70^	1 200 (IgG)	18–24 May	6.3	10 101 000	4 501 (28 May)	0.71 (0.57)

COVID-19: coronavirus disease 2019; Ig: immunoglobin; NR: not reported.

^a^ The seroprevalence was slightly lower in subsequent weeks.

^b^ The survey was done in Tbilisi, the capital city with a population 1.1 million. I could not retrieve the count of deaths in Tbilisi, but if more deaths happened in Tbilisi, then the infection fatality rate may be higher, but still < 0.1%.

^c^ Assuming a seroprevalence of 2.5%.

Notes: These are countries for which no eligible studies were retrieved in the literature search. The results of these studies have been announced to the press and/or in preliminary reports, but are not yet peer reviewed and published.

**Fig. 3:**
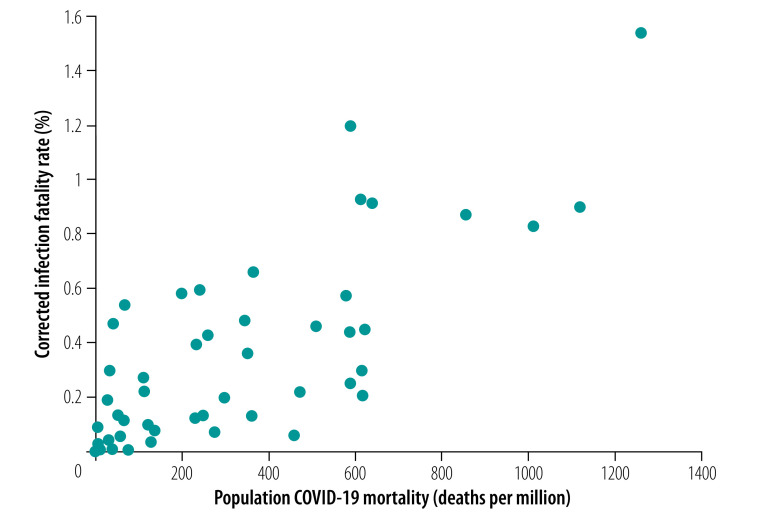
Corrected estimates of COVID-19 infection fatality rate in each location plotted against COVID-19 cumulative deaths per million as of September 12 2020 in that location

The median infection fatality rate across all 51 locations was 0.27% (corrected 0.23%). Most data came from locations with high death tolls from COVID-19 and 32 of the locations had a population mortality rate (COVID-19 deaths per million population) higher than the global average (118 deaths from COVID-19 per million as of 12 September 2020;^79^ Fig. 3). Uncorrected estimates of the infection fatality rate of COVID-19 ranged from 0.01% to 0.67% (median 0.10%) across the 19 locations with a population mortality rate for COVID-19 lower than the global average, from 0.07% to 0.73% (median 0.20%) across 17 locations with population mortality rate higher than the global average but lower than 500 COVID-19 deaths per million, and from 0.20% to 1.63% (median 0.71%) across 15 locations with more than 500 COVID-19 deaths per million. The corrected estimates of the median infection fatality rate were 0.09%, 0.20% and 0.57%, respectively, for the three location groups.

COVID-19: coronavirus disease 2019.

Notes: Locations are defined at the level of countries, except for the United Kingdom of Great Britain and Northern Ireland where they are defined by jurisdiction, United States of America (USA) are defined at the level of states and China is separated into Wuhan and non-Wuhan areas. Included locations are: Afghanistan; Argentina; Belgium; Brazil; Canada; Chile; China (non-Wuhan and Wuhan); Croatia; Czechia; Denmark; Faroe Islands; Finland; France; Georgia; Germany; Greece; Hungary; Iceland; India; Iran (Islamic Republic of); Israel; Italy; Japan; Kenya; Luxembourg; Netherlands; Pakistan; Qatar; Republic of Korea; Russian Federation; Slovenia; Spain; Sweden; Switzerland; United Kingdom (England, Scotland); and USA (California, Connecticut, Florida, Georgia, Idaho, Indiana, Louisiana, Minnesota, Missouri, New York, Pennsylvania, Rhode Island, Utah, Washington). When several infection fatality rate estimates were available from multiple studies for a location, the sample size-weighted mean is used. One outlier location with very high deaths per million population (1702 for New York) is not shown.

For people younger than 70 years old, the infection fatality rate of COVID-19 across 40 locations with available data ranged from 0.00% to 0.31% (median 0.05%); the corrected values were similar. 

## Discussion

The infection fatality rate is not a fixed physical constant and it can vary substantially across locations, depending on the population structure, the case-mix of infected and deceased individuals and other, local factors. The studies analysed here represent 82 different estimates of the infection fatality rate of COVID-19, but they are not fully representative of all countries and locations around the world. Most of the studies are from locations with overall COVID-19 mortality rates that are higher than the global average. The inferred median infection fatality rate in locations with a COVID-19 mortality rate lower than the global average is low (0.09%). If one could sample equally from all locations globally, the median infection fatality rate might even be substantially lower than the 0.23% observed in my analysis.

COVID-19 has a very steep age gradient for risk of death.^80^ Moreover, in European countries that have had large numbers of cases and deaths^81^, and in the USA^82^, many, and in some cases most, deaths occurred in nursing homes. Locations with many nursing home deaths may have high estimates of the infection fatality rate, but the infection fatality rate would still be low among non-elderly, non-debilitated people. 

Within China, the much higher infection fatality rate estimates in Wuhan compared with other areas of the country may reflect widespread nosocomial infections,^83^ as well as unfamiliarity with how to manage the infection as the first location that had to deal with COVID-19. The very many deaths in nursing homes, nosocomial infections and overwhelmed hospitals may also explain the high number of fatalities in specific locations in Italy^84^ and New York and neighbouring states.^23^^,^^27^^,^^35^^,^^56^ Poor decisions (e.g. sending COVID-19 patients to nursing homes), poor management (e.g. unnecessary mechanical ventilation and hydroxychloroquine) may also have contributed to worse outcomes. High levels of congestion (e.g. in busy public transport systems) may also have exposed many people to high infectious loads and, thus, perhaps more severe disease. A more aggressive viral clade has also been speculated.^85^ The infection fatality rate may be very high among disadvantaged populations and in settings with a combination of factors predisposing to higher fatalities.^37^

Very low infection fatality rates seem common in Asian countries.^8^^,^^11^^,^^29^^,^^48^^,^^49^^,^^51^^,^^59^^,^^61^^,^^67^ A younger population in these countries (excluding Japan), previous immunity from exposure to other coronaviruses, genetic differences, hygiene etiquette, lower infectious load and other unknown factors may explain these low rates. The infection fatality rate is low also in low-income countries in both Asia and Africa,^44^^,^^49^^,^^66^^,^^67^ perhaps reflecting the young age structure. However, comorbidities, poverty, frailty (e.g. malnutrition) and congested urban living circumstances may have an adverse effect on risk and thus increase infection fatality rate.

Antibody titres may decline with time^10^^,^^28^^,^^32^^,^^86^^,^^87^ and this would give falsely low prevalence estimates. I considered the maximum seroprevalence estimate when multiple repeated measurements at different time points were available, but even then some of this decline cannot be fully accounted for. With four exceptions,^10^^,^^28^^,^^32^^,^^51^ the maximum seroprevalence value was at the latest time point.

Positive controls for the antibody assays used were typically symptomatic patients with positive polymerase chain reaction tests. Symptomatic patients may be more likely to develop antibodies.^87^^–^^91^ Since seroprevalence studies specifically try to reveal undiagnosed asymptomatic and mildly symptomatic infections, a lower sensitivity for these mild infections could lead to substantial underestimates of the number of infected people and overestimates of the inferred infection fatality rate.

A main issue with seroprevalence studies is whether they offer a representative picture of the population in the assessed region. A generic problem is that vulnerable people at high risk of infection and/or death may be more difficult to recruit in survey-type studies. COVID-19 infection is particularly widespread and/or lethal in nursing homes, in homeless people, in prisons and in disadvantaged minorities.^92^ Most of these populations are very difficult, or even impossible, to reach and sample and they are probably under-represented to various degrees (or even entirely missed) in surveys. This sampling obstacle would result in underestimating the seroprevalence and overestimating infection fatality rate.

In principle, adjusted seroprevalence values may be closer to the true estimate, but the adjustments show that each study alone may have unavoidable uncertainty and fluctuation, depending on the type of analysis chosen. Furthermore, my corrected infection fatality rate estimates try to account for undercounting of infected people when not all three antibodies (IgG, IgM and IgA) were assessed. However, the magnitude of the correction is uncertain and may vary in different circumstances. An unknown proportion of people may have responded to the virus using immune mechanisms (mucosal, innate, cellular) without generating any detectable serum antibodies.^93^^–^^97^

A limitation of this analysis is that several studies included have not yet been fully peer-reviewed and some are still ongoing. Moreover, despite efforts made by seroprevalence studies to generate estimates applicable to the general population, representativeness is difficult to ensure, even for the most rigorous studies and despite adjustments made. Estimating a single infection fatality rate value for a whole country or state can be misleading, when there is often huge variation in the population mixing patterns and pockets of high or low mortality. Furthermore, many studies have evaluated people within restricted age ranges, and the age groups that are not included may differ in seroprevalence. Statistically significant, modest differences in seroprevalence across some age groups have been observed in several studies.^10^^,^^13^^,^^15^^,^^23^^,^^27^^,^^36^^,^^38^ Lower values have been seen in young children and higher values in adolescents and young adults, but these patterns are inconsistent and not strong enough to suggest that major differences are incurred by extrapolating across age groups.

Acknowledging these limitations, based on the currently available data, one may project that over half a billion people have been infected as of 12 September 2020, far more than the approximately 29 million documented laboratory-confirmed cases. Most locations probably have an infection fatality rate less than 0.20% and with appropriate, precise non-pharmacological measures that selectively try to protect high-risk vulnerable populations and settings, the infection fatality rate may be brought even lower.
